# From words to phrases: neural basis of social event semantic composition

**DOI:** 10.1007/s00429-022-02465-2

**Published:** 2022-02-20

**Authors:** Huichao Yang, Yanchao Bi

**Affiliations:** 1 State Key Laboratory of Cognitive Neuroscience and Learning & IDG/McGovern Institute for Brain Research, Beijing Normal University, Beijing, 100875 China; 2 Beijing Key Laboratory of Brain Imaging and Connectomics, Beijing Normal University, Beijing, 100875 China; 3 Chinese Institute for Brain Research, Beijing, 102206 China; 4 School of Systems Science, Beijing Normal University, Beijing, 100875 China

**Keywords:** Semantic composition, Social event, Semantic domain organization, Phrase comprehension, fMRI

## Abstract

Events are typically composed of at least actions and entities. Both actions and entities have been shown to be represented by neural structures respecting domain organizations in the brain, including those of social/animate (face and body; person-directed action) versus inanimate (man-made object or tool; object-directed action) concepts. It is unclear whether the brain combines actions and entities into events in a (relative) domain-specific fashion or via domain-general mechanisms in regions that have been shown to support semantic and syntactic composition. We tested these hypotheses in a functional magnetic resonance imaging experiment where two domains of verb-noun event phrases (social-person versus manipulation-artifact, e.g., “hug mother” versus “fold napkin”) and their component words were contrasted. We found a series of brain region supporting social-composition effects more strongly than the manipulation phrase composition—the bilateral inferior occipital gyrus (IOG), inferior temporal gyrus (ITG) and anterior temporal lobe (ATL)—which either showed stronger activation strength tested by univariate contrast, stronger content representation tested by representation similarity analysis, or stronger relationship between the neural activation patterns of phrases and synthesis (additive and multiplication) of the neural activity patterns of the word constituents. No regions were observed showing evidence of phrase composition for both domains or stronger effects of manipulation phrases. These findings highlight the roles of the visual cortex and ATL in social event compositions, suggesting a domain-preferring, rather than domain-general, mechanisms of verbal event composition.

## Introduction

Events are typically constituted by actions and entities, which have been shown to be supported by (partly) different brain networks (e.g., Vigliocco et al. 2011; Yang et al. 2017). Intriguingly, domain-preferential representations both for actions (e.g., social/human-directed and manipulation/object-directed; Wurm et al. 2017; Yang et al. 2020) and objects (e.g., animate and inanimate; Bi et al. 2016; Caramazza and Shelton 1998; Kanwisher 2010; Konkle and Caramazza 2013; Kriegeskorte et al. 2008b) have been reported as a salient neural organization principle. Does the brain also respect this domain organization in event composition (e.g., social-person constructions such as “hug mother” or manipulation-artifact constructions such as “fold napkin”)?

Previous studies of conceptual combination have endeavored to uncover the neural basis of understanding multiword phrases/sentences (see review in Frankland and Greene 2020) and understanding multi-object scenes (Baeck et al. 2013; Baldassano et al. 2017; Kaiser and Peelen 2018; Walbrin and Koldewyn 2019). Using language, mostly with adjective–noun phrases (e.g., red boat), researchers found that the bilateral angular gyrus (AG; Bemis and Pylkkänen 2013; Forgács et al. 2012; Graves et al. 2010; Lin et al. 2020; Pallier et al. 2011; Price et al. 2015; Price et al. 2016) and anterior temporal lobe (ATL; Bemis and Pylkkänen 2011; Pallier et al. 2011; Pylkkänen et al. 2014; Westerlund et al. 2015; Westerlund and Pylkkänen 2014) play important semantic composition roles in contrasts between meaningful and non-meaningful word pairs (e.g., activation for “red boat” > for “xkq boat” or “plaid jacket” > “moss pony”). Lin et al. (2020) examined verb–noun phrases and revealed two adjacently distributed subnetworks for sociality semantic and semantic combinations using similar univariate contrasts.

Neural compositional mechanisms have been tested in the context of nonverbal, multi-object scene representations, which tended to focus on the relationship between brain activities in higher-order visual regions to natural multi-object scene representations (e.g., image of a sofa in front of a television) and to object components (e.g., images of “sofa” and “television”), comparing linear (average) and nonlinear relationships. By testing such relationships at the neural level, these studies tested the neural implementations of different cognitive hypotheses about composition—independent versus interactive—assuming that additive neural composition implements a cognitive composition process where the two elements are relatively independently concatenated together (e.g., “superposition principle” in Smolensky and Legendre 2006; Mitchell and Lapata 2008), and multiplication neural composition implements an interactive process, such that the contribution of one component is scaled to its relevance to the other (e.g., see relevant discussions in Baron and Osherson 2011; Mitchell and Lapata 2008). Mixed patterns were observed, however, including effects of synthetic mean patterns (e.g., MacEvoy and Epstein 2009; Abassio and Papeo 2020); weighted average synthetic patterns (e.g., Baeck et al. 2013); nonlinear synthetic patterns (defined by shifting away from a simple average; e.g., Baldassanoo et al. 2017; Kaiser and Peelen 2018; Walbrin and Koldewyn 2019); or both multiplicative and additive synthetic patterns (Baron and Osherson 2011). While these studies provide interesting clues about the possible mechanisms in which compositions of neural representations may happen, mirroring the potential cognitive notions of verbal compositions (e.g., independent, context insensitive, Clark and Clark 1977 versus interactive, context sensitive, Keenan 1984; Murphy 1990; Chang et al. 2009; Mitchell and Lapata 2008), it is theoretically and empirically open whether the neural compositional mechanisms observed are general across modalities (e.g., pictures and words) and across domains (social-people versus manipulation-artifact).

In the present study, we tested specifically whether verbal semantic composition for event understanding was supported by domain preference (social action versus manipulation artifact) or domain-general neural mechanisms, using univariate contrast for activity strength analyses, multivariate representation similarity analysis (RSA) for information content analyses, and neural activity pattern correlation analyses between the phrase and the component word conditions for testing the neural compositional computation mechanisms. In an event-related design fMRI experiment, participants silently read two domains of verb-noun phrases (i.e., social-person constructions, such as “hug mother” and manipulation-artifact constructions such as “fold napkin”) and their component words (e.g., “hug”, “mother”, “fold”, “napkin”). Unmatched phrases (i.e., social-artifact such as “hug napkin” and manipulation-person constructions such as “fold mother”) were also included as control conditions. Regions showing phrase composition effects across both domains would indicate potential domain-general effects of composition, and those showing phrase composition effects in only one domain or more strongly in one domain would suggest domain-preference compositional effects for that domain.

## Materials and methods

### Participants

Twenty-seven (12 males; age 21.5 ± 2.7) right-handed healthy adults participated in this fMRI study. They all had normal or corrected-to-normal vision. Informed consent was provided by all participants, and the procedure was approved by the institutional review board of the State Key Laboratory of Cognitive Neuroscience and Learning, Beijing Normal University. All methods were performed in accordance with relevant named guidelines and regulations.

### Stimuli

The stimuli set contained 20 verb–noun phrases and 20 individual words that constituted those phrases (Table 1). The twenty individual words satisfied 2 social- and 2 manipulation-related conditions (5 in each condition): social action and person or manipulation action and artifact. The twenty phrases satisfied 2 matched and 2 unmatched conditions (5 in each condition): social-person and manipulation-artifact or social-artifact and manipulation-person. We considered a range of potential confounding variables across conditions (nonparametric statistical testing Kruskal–Wallis Test or Mann–Whitney Test was employed given the small sample size; Table 2). Specifically, component words were matched on visual complexity, frequency, and familiarity across the four conditions: number of strokes: *χ*^2^ = 0.271, *p* = 0.965; logarithm word frequency (words not included in the corpus were set as 0; Sun et al. 2018, http://www.chineselexicaldatabase.com): *χ*^2^ = 5.302, *p* = 0.151; familiarity ratings (1–7) from an independent group of 22 participants (6 males; age: 25.1 ± 3.1): *χ*^2^ = 1.885, *p* = 0.597. For phrases, we considered visual complexity and three measures of composition strength: bigram frequencies, transition probabilities, and plausibility ratings. Bigram frequencies (as computed in Price et al. 2015; log(*x*), phrases not included in the corpus were set as 0) and transition probabilities (frequency of phrase/frequency of the verb) were obtained using Chinese Web Google *n*-gram corpus (https://catalog.ldc.upenn.edu/LDC2010T06; Liu et al. 2010); plausibility ratings were obtained from the same group of participants that did the familiarity ratings. The visual complexity was well matched among the four phrase conditions (mean stroke number: *χ*^2^ = 0.202, *p* = 0.977). The three composition strength measures varied in alignment with our design (matched > unmatched; no difference within matched or within unmatched): the composition strength of matched phrases was significantly higher than that of unmatched phrases across different measures (*U*s ≤ 10, *p*s ≤ 0.002). There were no significant differences between the two matched (e.g., “hug mother” versus “fold napkin”; *U*s ≥ 5, *p*s ≥ 0.151) or the two unmatched conditions (e.g., “hug napkin” versus “fold mother”; *U*s = 7.5, *p*s = 0.310). In addition, each word or phrase had a homophone pseudoword that was used as a catch trial during the experiment.

**Table 1:** Stimuli set used in the experiment

	Social action	Person	Manipulation action	Artifact
Individual words	亲吻 Kiss 搀扶 Support somebody with hand 拥抱 Hug 追赶 Chase 目送 Gaze after	恋人 Lover 病患 Patient 母亲 Mother 强盗 Robber 朋友 Friend	穿上 Put on 折叠 Fold 转动 Turn 翻开 Open 点击 Click	雨衣 Raincoat 纸巾 Napkin 魔方 Cube 相册 Photo album 鼠标 Mouse

	Matched	Unmatched		
	Social-person	Manipulation-artifact	Manipulation-person	Social-artifact
Phrases	亲吻恋人 Kiss lover 搀扶病患 Support patient with hand 拥抱母亲 Hug mother 追赶强盗 Chase robber 目送朋友 Gaze after friend	穿上雨衣 Put on raincoat 折叠纸巾 Fold napkin 转动魔方 Turn cube 翻开相册 Open photo album 点击鼠标 Click mouse	穿上恋人 Put on lover 折叠病患 Fold patient 转动母亲 Turn mother 翻开强盗 Open robber 点击朋友 Click friend	目送雨衣 Gaze after raincoat 搀扶纸巾 Support napkin with hand 拥抱魔方 Hug cube 追赶相册 Chase photo album 亲吻鼠标 Kiss mouse

**Table 2:** Lexical–semantic variables of each condition

	Condition	Example	Number of stroke	Log frequency (per million)	Familiarity rating score
Component words	Social action	拥抱(hug)	16.80 ± 2.17	0.72 ± 0.71	6.61 ± 0.16
	Person	母亲(mother)	16.40 ± 5.22	1.73 ± 1.15	6.55 ± 0.37
	Manipulation action	折叠(fold)	16.40 ± 4.34	0.40 ± 0.37	6.75 ± 0.07
	Artifact	纸巾(napkin)	16.80 ± 5.93	0.97 ± 0.61	6.69 ± 0.18

	Condition	Example	Number of stroke	Bigram frequency	Transition probability	Plausibility rating score
Phrases	Social-person	拥抱母亲(hug mother)	33.20 ± 7.29	2.49 ± 1.53	5.96 × 10^–4^ ± 6.17 × 10^–4^	4.83 ± 0.17
	Manipulation-artifact	折叠纸巾(fold napkin)	33.20 ± 5.02	3.97 ± 1.22	1.52 × 10^–3^ ± 1.36 × 10^–3^	4.87 ± 0.22
	Manipulation-person	折叠病患(fold patient)	32.80 ± 9.52	1.01 ± 1.50	2.88 × 10^–6^ ± 4.34 × 10^–6^	1.23 ± 0.19
	Social-artifact	拥抱魔方(hug cube)	33.60 ± 5.32	0.00 ± 0.00	0.00 ± 0.00	1.44 ± 0.40

### Experimental design

#### Procedures

**Fig. 1:**
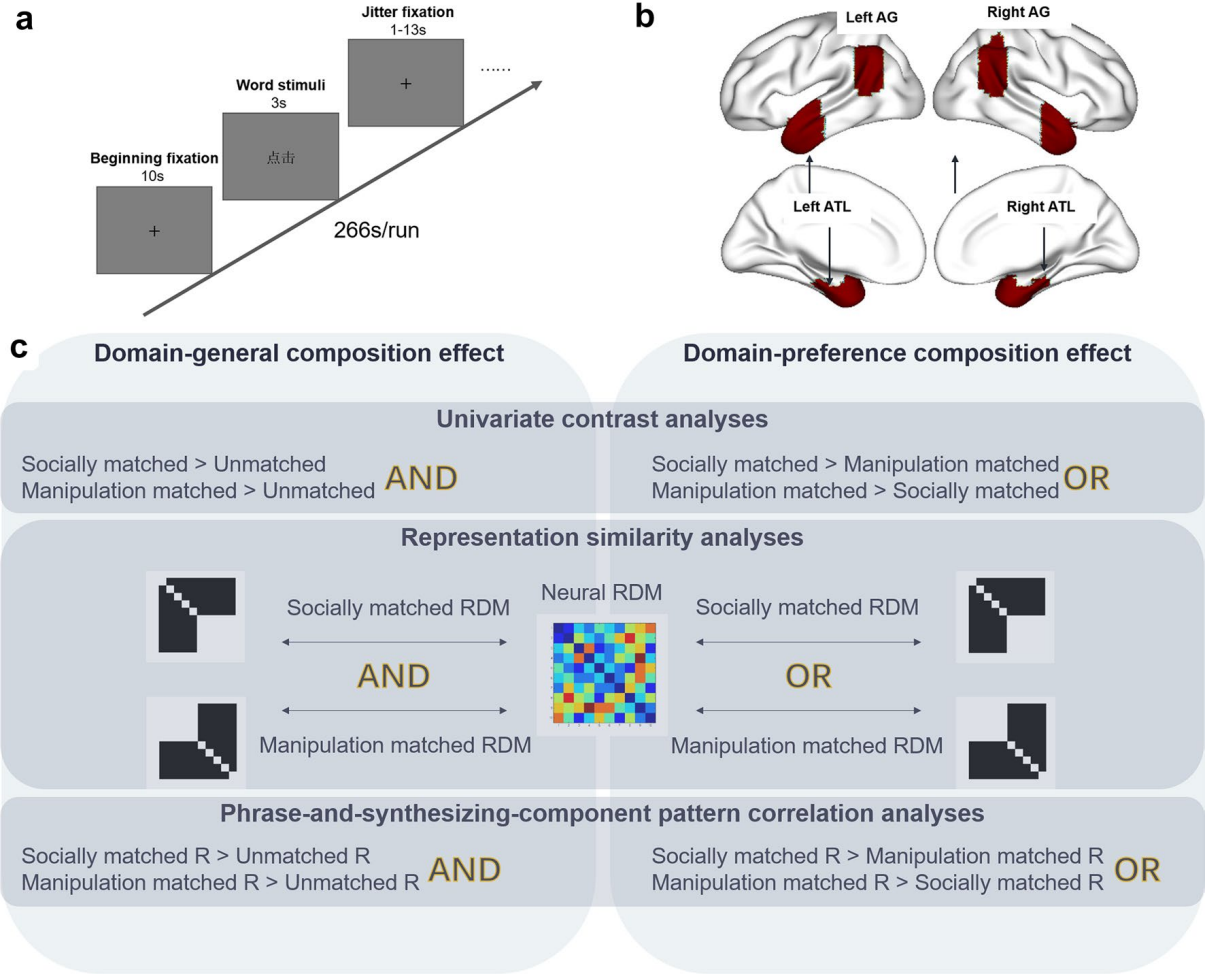
Experimental design. **a** Procedures in a run. Each trial consisted of 3 s stimuli presentation and a 1 to 13 s jitter fixation. **b** Spatial location of the bilateral ATL and bilateral AG, which were defined by the Harvard–Oxford Atlas. **c** Hypotheses testing of domain-general and domain-preference composition effects across three analyses

This study had an event-related design. Each participant completed 8 or 10 runs (seven of the twenty-seven participants completed 10 runs) in all: half were individual word runs and half were phrase runs. As mentioned above, all of the phrases were formed by individual words. All participants completed individual word runs before completing phrasing runs to prevent phrase processing from influencing individual word processing. That is, if participants read “hug mother” before “mother”, they may have automatically thought of “hug mother”. Each run consisted of a 10 s beginning fixation period, followed by 48 trials in the different conditions (each of 20 stimuli was repeated two times, and 8 pseudoword catch trials were included). Each trial consisted of 3 s stimuli and a jitter fixation of at least 1 s. Each run lasted 266 s (Fig. 1a). The order of stimuli conditions and null events were optimized using optseq software (Dale 1999; http://surfer.nmr.mgh.harvard.edu/optseq/) and were counterbalanced across runs and participants. The trial order within each condition was randomized for each participant. The experimental procedure was presented using Eprime 2.0 (https://pstnet.com/products/e-prime/).

#### Tasks

The participants were asked to complete a phonetic one-back task. Specifically, they read each word silently; when they read a word that had the same pronunciation as the prior word, they needed to press a button with their left index finger. All of the trials that needed responses (homophone-one-back catch trials) were pseudoword. To prevent participants from pressing the button as soon as they read a pseudoword, we also included half pseudoword trials that did not require a response (i.e., not being homophone to the previous word).

### Image acquisition and preprocessing

The scan data were collected with a Siemens Trio Tim 3 T scanner at the Brain Imaging Center of Beijing Normal University. All participants underwent structural image scanning first, followed by functional image scanning. T1-weighted three-dimensional magnetization-prepared rapid gradient echo (3D-MPRAGE) images were collected in 144 sagittal slices (repetition time (TR) = 2530 ms, echo time (TE) = 3.39 ms, flip angle = 7°, slice thickness = 1.3 mm, slice in-place resolution = 1.3 × 1.0 mm^2^, and field of view (FOV) = 256 × 256 mm^2^). Blood oxygen level-dependent (BOLD) fMRI images were obtained using an echo-planar imaging (EPI) sequence in 33 axial slices (TR = 2000 ms, TE = 30 ms, flip angle = 90°, slice thickness = 3.5 mm, slice gap = 0.7 mm, slice in-place resolution = 3 × 3 mm^2^, and FOV = 64 × 64 mm^2^).

The magnetic resonance imaging (MRI) data were preprocessed by Statistical Parametric Mapping software (SPM12, http://www.fil.ion.ucl.ac.uk/spm/software/spm12/). The first 5 volumes of each run were discarded. Then, slice-timing and 3-D motion correction were performed. No participant showed excessive head motion (< 2 mm or 2°). T1 images of each participant were co-registered to their mean functional image. Functional images were then normalized to Montreal Neurological Institute (MNI) space using the segmented T1 images and resampled to 3 × 3 × 3 mm^3^. The resulting unsmoothed data were used to perform multivariate pattern analyses. Spatial smoothing was applied with a 6 mm full-width at half-maximum (FWHM) Gaussian kernel for univariate analyses.

### Domain-general or domain-preference phrase composition effect estimation

Event phrase semantic composition effects were considered in three types of analyses: Univariate contrast analyses were conducted to reveal brain regions showing preferring sensitivity to either domains of phrases; RSA was conducted to examine whether the neural activity pattern is sensitive to either or both domains’ memberships; Phrase-and-synthesizing-component pattern correlation analyses focused on uncovering whether and how the neural activity patterns of the phrases could be composed from the neural activity patterns of their constituent words. Across all three analyses, potential domain-general and domain-preferring effects are considered: regions showing phrase effects across both domains would indicate potential domain-general phrase composition effects, and those showing phrase effects in only one domain or more significantly in one domain would suggest domain-preferring phrase compositional effects for that domain (Fig. 1c).

#### Univariate contrast analyses

First, we conducted whole-brain univariate contrast analyses. Brain regions showing stronger activations relative to unmatched control conditions across both domains (i.e., socially matched > unmatched ∩ manipulation matched > unmatched) would indicate domain-general phrase composition effect, and those showing stronger activations for one domain relative to another (i.e., socially matched > manipulation matched or manipulation matched > socially matched) indicate domain-preferring phrase composition effects. We also compared the social and manipulation conditions of individual words to test whether the domain-preference composition effects could be explained by effects of single words. Phrase runs and individual word runs were analyzed in parallel but separately. Then, the smoothed functional images were entered into the general linear model (GLM). Eleven predictors were included: the four conditions of phrase or individual word runs, one merged catch trial condition and six motion parameters. The high-pass filter cutoff (128 s) was set as the default. A lenient threshold of the implicit mask (0.01) was used to ensure coverage of the anterior temporal lobe. Contrasts between social-person or manipulation-artifact phrases versus the two across-category unmatched control conditions (e.g., “hug mother” or “fold napkin” versus “hug napkin” and “fold mother”), social-person versus manipulation-artifact phrases (e.g., “hug mother” versus “fold napkin”) and social words (i.e., social actions and persons) versus manipulation words (i.e., manipulation actions and artifacts) were built and computed for each participant. One-sample t test analyses were performed to compare the mean activation across participants with zero in the second-level analyses (threshold set as voxel level *p* < 0.001; cluster extent familywise error (FWE) *p* < 0.05). A gray matter mask (probability higher than 0.4 in the SPM5 gray matter template) that excluded the cerebellar regions (#91-#116 of the Automated Anatomical Labeling template) was used in this step. All of the surface brain maps in the present study were visualized with the BrainNet Viewer (Xia et al. 2013; http://www.nitrc.org/projects/bnv/).

#### Anatomically defined region of interest (ROI)

Bilateral ATL and AG were reported to have important roles in semantic and syntactic composition according to previous findings of multiword combinations (see “Introduction”). We defined four ROIs using the anatomical template of the Harvard–Oxford Atlas (probability > 0.2; Fig. 1b) to test whether participants combined the actions and entities into events in a domain-preferential way or via domain-general representation. The bilateral AG was region #20 in the template. The bilateral ATL was defined as the union set of the temporal pole (#7), the anterior superior temporal gyrus (#8), the anterior middle temporal gyrus (#10), the anterior inferior temporal gyrus (#13), the anterior temporal fusiform cortex (#33), and the anterior parahippocampal gyrus (#36). Left and right ROIs were saved separately and then resampled to a 3 × 3 × 3 mm^3^ space (same with preprocessed functional images).

#### RSA

To test whether the multivariate activation patterns across voxels of different regions represent different domains of combined content, we performed RSA (Kriegeskorte et al. 2008a) to evaluate the individual word runs and phrase runs. Both the theoretical representation dissimilarity matrix (RDM) and brain RDM were item-based matrices. That is, each element represented the dissimilarity between two words or phrases (e.g., “fold napkin” and “click mouse”). The two theoretical model RDMs of the matched phrases are shown in Fig. 3a: the socially matched RDM was grouped by the social-person phrases (e.g., “hug mother”), and the manipulation-matched RDM was grouped by the manipulation-artifact phrases (e.g., “fold napkin”). The two RDMs were negatively correlated (Spearman’s *ρ* = − 0.286). We hypothesized that if a region represent event phrases in a domain-general way, it would show significant positive correlation with both the socially matched RDM and the manipulation-matched RDM (social phrases are more similar to each other; manipulation phrases are more similar to each other). If a region represent relatively specifically meanings of social or manipulation event phrases, it would correlate with the theoretical RDM of the corresponding domain. The two theoretical model RDMs of individual words are shown in Fig. 3b in a similar manner.

To obtain neural RDMs, we first created GLMs at the item level. The unsmoothed preprocessed functional images were entered into the GLM to obtain the activation pattern across voxels. Twenty-seven predictors were included in the GLM: twenty phrase or single-word conditions, one merged catch trial condition and six motion parameters. Due to the high noise of beta estimates (Misaki et al. 2010), the *t *value images of each phrase were calculated to aid the construction of the neural RDM. Each element in the neural matrix measured the dissimilarity (1 − Spearman’s *ρ*) between the activation patterns of two phrases or single words across voxels in a given brain region. Then, we calculated the Spearman’s correlation between the neural RDM and the theoretical model RDMs. RSA was implemented at both the whole-brain searchlight level and ROI level.

Whole-brain searchlight RSA was implemented within the gray matter mask excluding cerebellar regions (in the same manner as for the univariate contrast analyses). A 10-mm-radius sphere (171 voxels) was built for each voxel. Then, we extracted the activity patterns of these voxels across different words or phrases to build the neural RDM. The neural RDM of each voxel was correlated with the model RDMs and Fisher-transformed. For each model, each voxel had a correlation value, resulting in a similarity whole-brain map for each participant. The correlation maps were spatially smoothed with a 6 mm FWHM Gaussian kernel. One-sample *t *test analyses were applied to compare the mean correlation across participants with zero using the permutation-based nonparametric method (Nichols and Holmes 2002) with SnPM 13 (http://warwick.ac.uk/snpm; threshold set as voxel level *p* < 0.001; cluster extent FWE *p* < 0.05). For the ROI-level RSA, we extracted the activity patterns within a specific ROI across different words or phrases to construct the neural RDM of each ROI. One-sample *t *test analyses across participants were performed in SPSS 20.0 (threshold set as two-tailed, *p* < 0.05). Other procedures were the same as those used in the whole-brain searchlight analysis.

#### Pattern correlation analyses between phrases and the combination of their word components

To reveal the neural compositional mechanisms of individual words into phrases (events), we applied multiplicative (i.e., multiplying the neural vectors of component words) and additive models (i.e., adding the neural vectors of component words) to the activation patterns of the individual words. Then, we computed correlations between the actual observed activation patterns of phrases and those synthetic neural patterns based on constituents. A flowchart of these phrase-and-synthesizing-components pattern correlation analyses is shown in Fig. 4a. Both whole-brain searchlight analyses and ROI-level analyses were conducted. Specifically, we extracted the activation pattern in response to each stimulus (each individual word and each phrase) within a 10-mm-radius sphere of a voxel or a specific ROI. For each word pair that constituted a phrase (e.g., “hug” and “mother”), we obtained the estimated synthetic activation patterns by two basic composition models—additive and multiplicative. Next, Spearman’s correlation between the estimated synthetic pattern and the real pattern in response to each phrase was calculated and then Fisher-transformed. The average correlation coefficients of the five examples of socially or manipulation-matched phrases were labeled as socially matched R and manipulation-matched R, respectively. The unmatched phrases were constructed across the two domains (social and manipulation) and did not belong to either one; therefore, we merged the ten examples of unmatched phrases and labeled the averaged correlation coefficient as unmatched R. If a region meaningfully compose event phrases in a domain-general way, it would show stronger correlations in both matched domains relative to the unmatched conditions. If a region undergoes composition in a domain-preferring manner, it would show stronger correlations with one domain relative to the other (lowest panel of Fig. 1c). Comparison between social- or manipulation-matched R and unmatched R and comparison between the two domains of matched Rs were implemented by SnPM in whole-brain analyses and by SPSS 20.0 in ROI analyses.

## Results

### Univariate contrast between social-person matched phrases and manipulation-artifact matched phrases

**Fig. 2:**
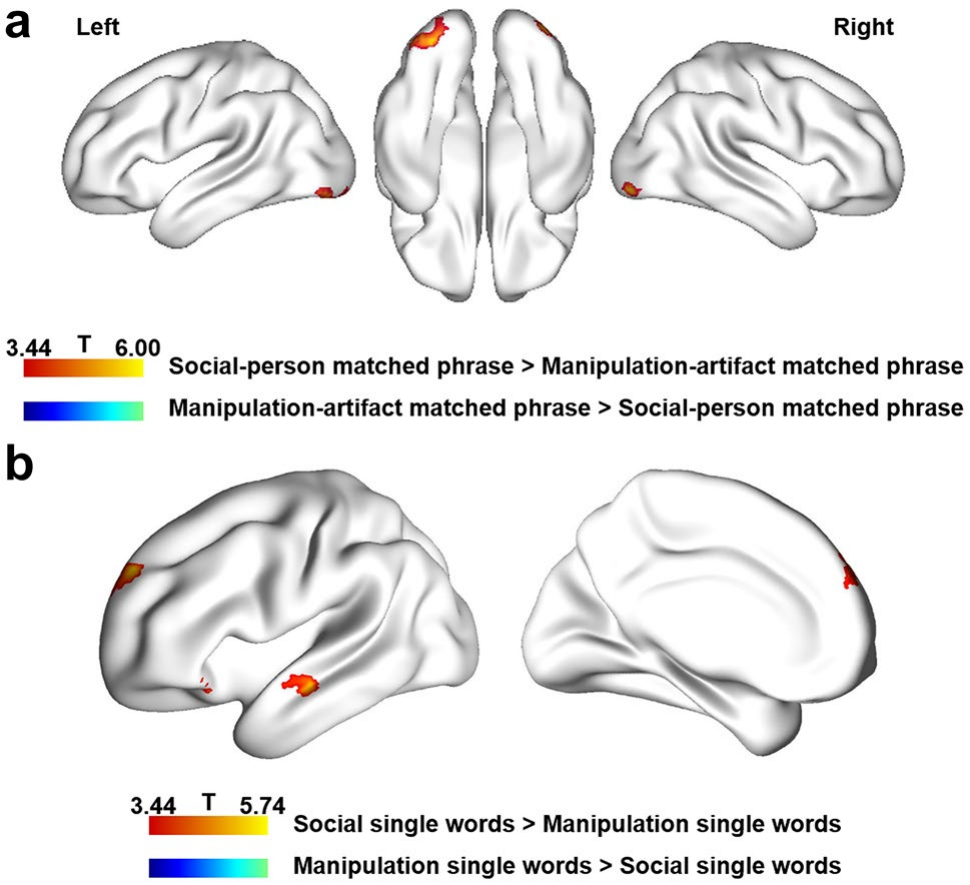
Whole-brain univariate analysis results. **a** Whole-brain univariate analysis results contrasting social person meaningfully matched phrases (e.g., “hug mother”) and manipulation artifact meaningfully matched phrases (e.g., “fold napkin”). **b** Whole-brain univariate analysis results contrasting single words in the social condition (social actions and persons) versus single words in the manipulation condition (manipulation actions and artifacts). Both thresholds were set at voxel level *p* < 0.001, cluster-extent FWE corrected *p* < 0.05

The results of whole-brain univariate contrast between two domains of matched phrase conditions (e.g., “hug mother” versus “fold napkin”) are shown in Fig. 2a. Social-person phrases induced greater activation than manipulation-artifact phrases in the bilateral inferior occipital gyrus (peak MNI coordinates of the bilateral IOG: − 30, − 84, − 12; 27, − 90, − 6; threshold: voxel level *p* < 0.001; cluster extent FWE *p* < 0.05). No regions showed significantly greater reversed activation for manipulation-artifact phrases, or domain-general phrase composition effects (i.e., conjunction analyses of social-person phrases and manipulation-artifact phrases relative to the unmatched conditions), under the same thresholds. When comparing the two domains of individual words, i.e., social actions and persons (e.g., “hug”, “mother”) versus manipulation actions and artifacts (e.g., “fold”, “napkin”), three clusters located in the left inferior orbital frontal gyrus, left anterior middle temporal gyrus and left superior frontal gyrus showed stronger activation in the social conditions than in the manipulation condition (Fig. 2b; threshold: voxel level *p* < 0.001; cluster extent FWE *p* < 0.05). The bilateral IOG showing social phrases preference (relative to manipulation phrases) did not indicate social preference in the single-word condition under the same whole-brain analysis threshold, but it demonstrated a significant preference for social words in ROI-level analyses (left IOG: *t*(26) = 2.841; *p* = 0.009; right IOG: *t*(26) = 3.432; *p* = 0.002).

### RSA results of socially matched and manipulation-matched phrases

**Fig. 3:**
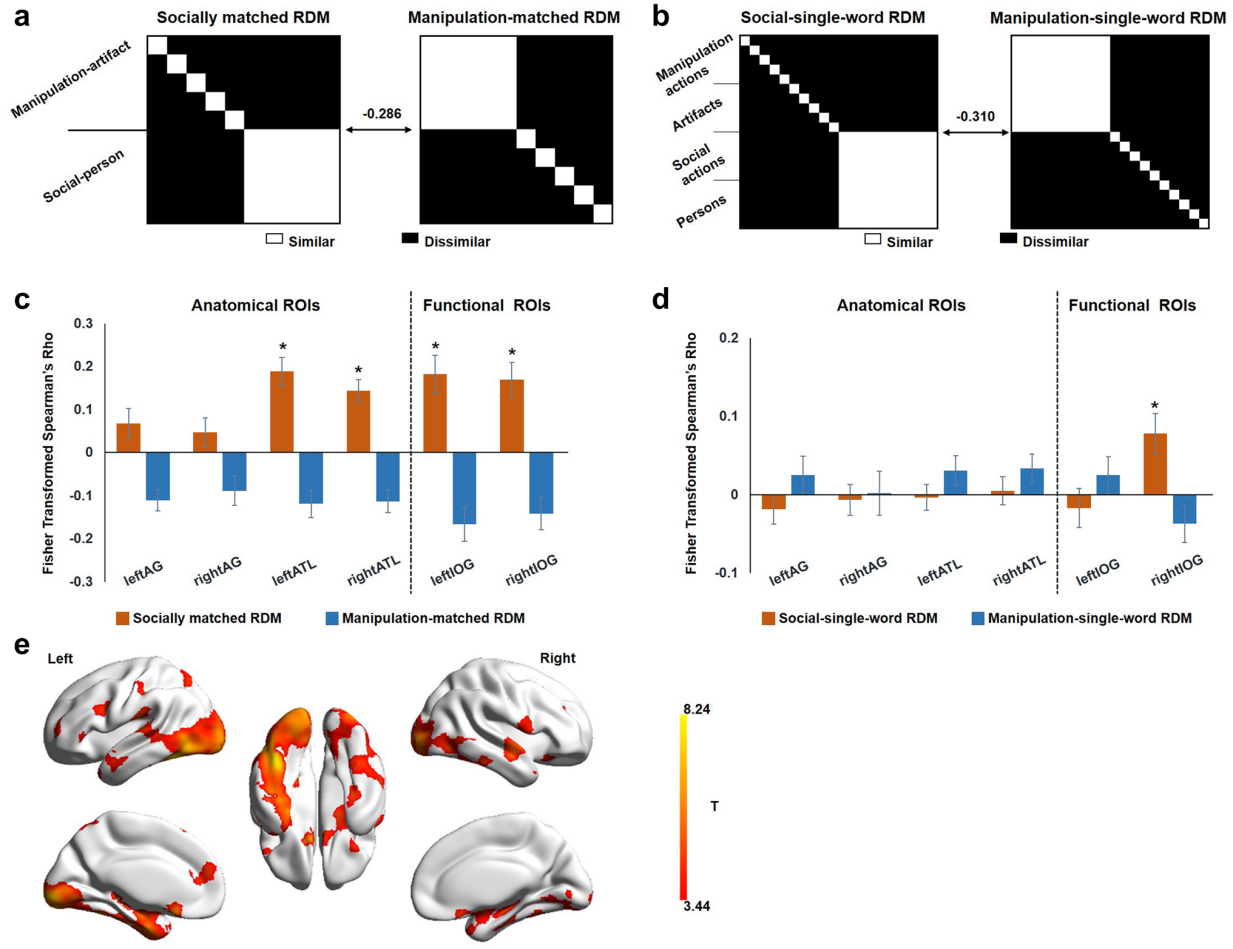
RSA results. **a** Theoretical RDM of social- and manipulation-matched phrases. **b** Theoretical RDM of single words in the social and manipulation conditions. **c** ROI-level RSA results for phrases. **d** ROI-level RSA results for single words. The * above bars indicate a significant difference from zero (two-tailed, *p* < 0.05). The four anatomical ROIs were the bilateral ATL and AG, which have been shown in the literature to support semantic and syntactic composition; two functional ROIs (bilateral IOG) were those showed domain preference to social-action-person phrases (see Fig. 2a). **e** Whole-brain RSA results of socially matched phrases. Brain regions showing significant positive correlations with socially matched RDMs (threshold: voxel level *p* < 0.001 and cluster extent FWE *p* < 0.05). No positive results were found for manipulation-matched phrases in these analyses

ROI and whole-brain searchlight RSA of multivariate activation patterns across voxels were performed to reveal brain regions representing different domains of combinations. Theoretical semantic RDMs were constructed by grouping phrases or words from the same domain together (Fig. 3a, b). Neural RDMs were obtained based on the activation pattern in a given brain region. There were two kinds of ROIs: four anatomical ROIs (bilateral AG and ATL) that have been previously shown in the literature to support semantic and syntactic composition and two functional ROIs (bilateral IOG) that showed domain preference for the social-person phrase (e.g., “hug mother”) obtained above in the univariate contrast. The neural RDMs of the bilateral ATL and bilateral IOG showed significant correlations with the socially matched RDM (Fig. 3c; *t*s > 4.055, *p*s < 4.053 × 10^–4^), indicating these regions are sensitive to social semantics such that social-person phrase pairs (e.g., “hug mother”–“kiss lover”) yielded more similar neural activity patterns here. No ROIs showed any trend of effects of manipulation-matched RDM. Among these ROIs, only the neural RDM of the right IOG showed significant correlations with the social-single-word RDM (Fig. 3d; *t*(26) = 2.996, *p* = 0.006). These results indicate that the right IOG represents social preference for both single words and phrases, while left IOG and bilateral ATL showed sensitivity to social event phrases only.

For the whole-brain searchlight analyses, brain regions positively correlated with the socially matched phrase RDM included the bilateral ATL, ventral temporal gyrus, occipital gyrus, middle frontal gyrus, left superior parietal lobe and superior medial frontal region (Fig. 3e; thresholds: voxel level *p* < 0.001; cluster extent FWE *p* < 0.05). No brain regions were found to have a significant positive correlation with the manipulation-matched RDM.

### Results of phrase-and-synthesizing-component pattern correlation analyses

**Fig. 4:**
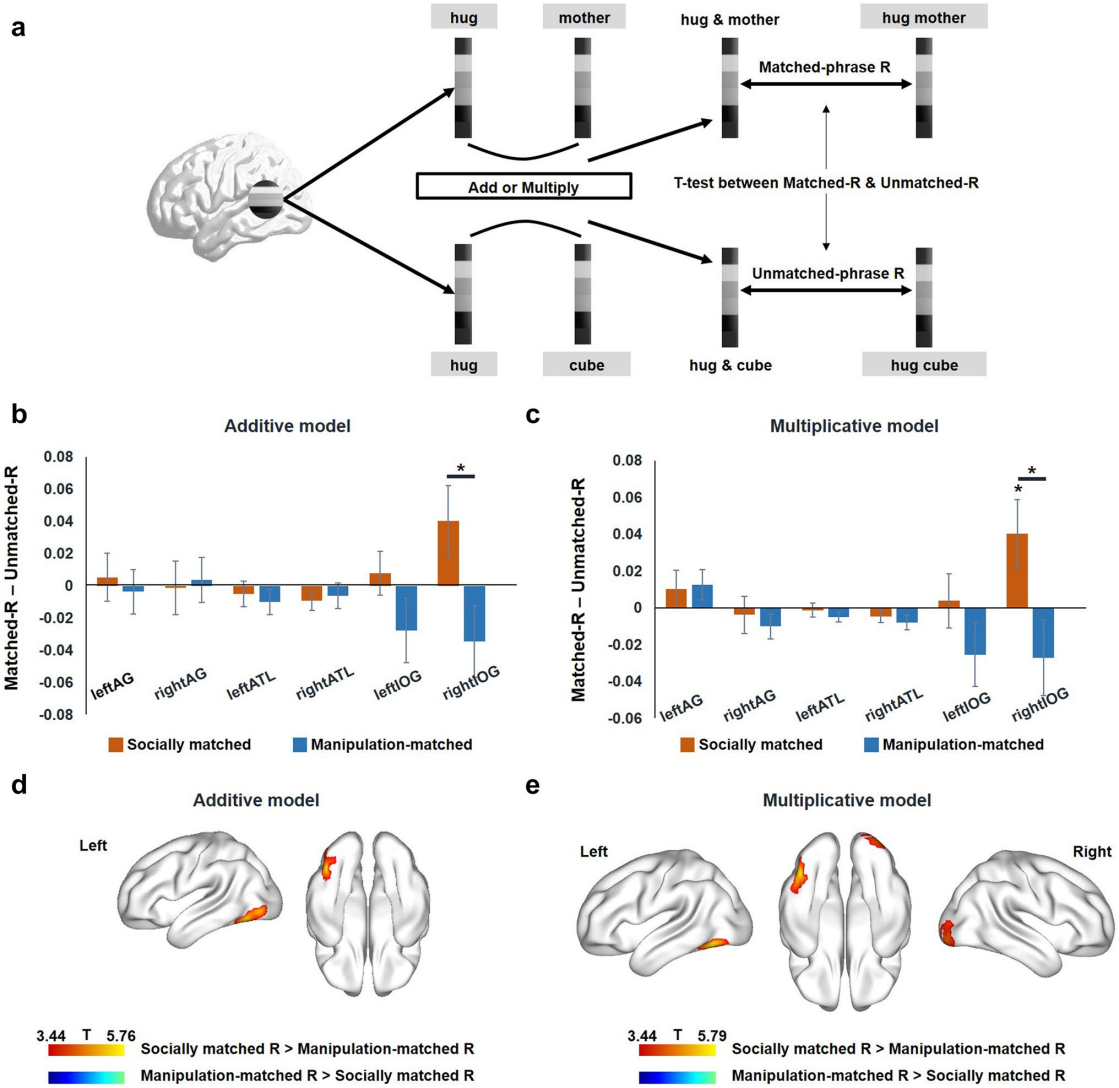
Results of phrase-and-synthesizing-components pattern correlation analyses. **a** A flowchart of phrase-and-synthesizing-components pattern correlation analyses, i.e., correlation between actual activation patterns observed in response to phrases and multiplicative or additive synthetic patterns observed in response to its corresponding component words. **b** Correlation between phrase activity patterns and synthetic-sum patterns observed in response to word components in the anatomical ROIs and functional ROIs. **c** Correlation between phrase activity patterns and synthetic-product patterns observed in response to word components in the anatomical ROIs and functional ROIs. Socially matched R means correlations between the observed activation patterns of social-action-person phrases and the synthesis of the activation patterns of the corresponding component words; manipulation-matched R means correlations between the observed activation patterns of manipulation-artifact phrases and the synthesis of the activation patterns of the corresponding component words. Unmatched-R means correlations between the observed activation patterns of the unmatched phrases and the synthesis of the activation patterns of their corresponding component words. The * above bars indicate a significant difference from zero (two-tailed, *p* < 0.05), and the * above black lines indicate a significant difference between the two conditions (two-tailed, *p* < 0.05). **d** Contrast between whole-brain maps of socially matched R and manipulation-matched R for the additive model (threshold: voxel level *p* < 0.001 and cluster extent FWE *p* < 0.05). **e** Contrast between whole-brain maps of socially matched R and manipulation-matched R for the multiplicative model (threshold: voxel level *p* < 0.001 and cluster extent FWE *p* < 0.05)

To further test the neural computational mechanisms in which single-word constituents are composed into phrases, we applied two basis composition models (multiplicative and additive) to test the relationship between the neural activation patterns between phrases and constituents. We computed correlations between the actual activation patterns of phrases and the multiplicative synthetic patterns or additive synthetic patterns of the corresponding component words. Specifically, the neural activation pattern across voxels in a given brain region is represented as a neural vector for each component word. We then performed either multiplication or addition on these two component word neural vectors, and computed the correlation between the resulting composite neural vector and the actual observed neural vector for phrase reading. These phrase-and-synthesizing-components correlations were compared between semantically meaningful (i.e., matched) phrases and unmatched phrases and between the two domains of matched phrases. Among the six ROIs (see above), the right IOG (Fig. 4b, c) showed a significantly higher correlation coefficient in the socially matched condition (e.g., “hug mother”) than in the unmatched conditions (e.g., “hug cube”; multiplicative model: *t*(26) = 2.191, *p* = 0.038; nonsignificant trend in the additive model: *t*(26) = 1.848, *p* = 0.076), and than in the manipulation-matched condition (e.g., “fold napkin”; additive: *t*(26) = 2.620, *p* = 0.014; multiplicative: *t*(26) = 2.543, *p* = 0.017), indicating that the neural responses of social phrases were significantly explained by both additive and multiplication mechanisms of the component words here. No significant differences were obtained in the other ROIs, or for the manipulation-matched condition in any of the ROIs (*t*s < 1.985, *p*s > 0.058).

Whole-brain searchlight analyses revealed that the left posterior inferior temporal gyrus (ITG; peak coordinate: − 42, − 63, − 18) showed higher phrase-and-synthesizing-components correlations in the socially matched condition than in the manipulation-matched condition for the additive model (Fig. 4d; threshold: voxel level *p* < 0.001; cluster extent FWE *p* < 0.05). The right IOG (peak coordinate: 30, − 93, − 9) and the left ITG (peak coordinate: − 42, − 63, − 15) showed higher phrase-and-synthesizing-components correlations in the socially matched condition than in the manipulation-matched condition for the multiplicative model (Fig. 4e; threshold: voxel level *p* < 0.001; cluster extent FWE *p* < 0.05). No brain regions showed higher phrase-and-synthesizing-components correlations across the two domains relative to unmatched condition in a domain-general way under the same threshold.

## Discussion

Through an experiment that included two domains of action-entity phrases describing events, i.e., social-action-person and manipulation-artifact phrases (“hug mother”; “fold napkin”), and their word components, we tested whether event phrases of multiple conceptual elements are processed in a domain-preference or domain-general manner. Across univariate, representation–similarity–analysis, and pattern–synthesis analysis, the following findings emerged. First, when participants processed socially matched phrases, such as “hug mother”, stronger activations were elicited in the bilateral IOG than those elicited by manipulation-matched phrases, such as “fold napkin”. Second, the right IOG and left posterior ITG showed stronger effects of actual “composition” for social event phrases—the activation patterns observed for the social-action-person phrases (e.g., “hug mother”) can be significantly predicted from synthesizing, by adding or multiplying, the neural activation patterns of the word components. Third, RSA revealed that the neural activity patterns of the bilateral IOG, broader IT and the bilateral ATL showed a significant domain-preference positive correlation with the socially matched RDM. We discuss these findings in the visual cortex and the ATL in turn below.

### Semantic composition of social events in the visual cortex

A set of regions in the visual cortex, especially IOG (extending to ITG), showed consistent sensitivity to social event (e.g., “hug mother”) phrase composition across multiple analyses, without similar effects for manipulation events (e.g., “fold napkin”): social phrases elicited stronger activation than manipulation phrases; the neural activity pattern is more similar among social phrases; and only for social phrases, the neural activity patterns can be significantly explained by synthesizing (addition and multiplication) of the neural vectors of the components.

Such robust finding in IOG (and ITG) is slightly unexpected given that this region is not part of the brain networks that are commonly considered to support social semantic processing (e.g., Zhang et al. 2021). Its implication in social processing, however, has actually been hinted in various contexts before. The bilateral IOG cluster we observed is adjacent the occipital face area, which show particular sensitivity to face/face part (reviews in Pitcher et al. 2011 and Wang and Olson 2018). Its involvement in high-level semantic social association functions beyond simple visual part features has been suggsted. It plays a causal role in face-job title associations, as shown by a study with transcranial magnetic stimulation (Eick et al. 2020); it is more strongly activated by animations of two triangles involving theory of mind than random animations. The other face/body-related regions in the ventral visual cortex (fusiform face area and extrastriate body area), close to the ITG cluster, we observed sensitive to social verbal semantic composition in the RSA and pattern-synthesis analyses have been shown to be also sensitive to social event composition of nonverbal stimuli, although the synthesis mechanisms varied across studies (synthetic mean, Abassi and Papeo 2020; nonlinear synthetic pattern, Walbrin and Koldewyn 2019). These findings together suggest that these visual areas are engaged in social event semantic composition across multiple modalities (i.e., both picture, video, and language). Whether this pattern reflects certain common combination mechanisms for multimodal social semantics or a co-activation of the visual imageries associated with verbal social event semantic composition warrants further investigation. Also open is the specific compositional mechanism, as we found that both addition and multiplication operations of the neural representations had comparably significant effect here, without being able to distinguish between potential independent versus interactive mechanisms of composition (see similar findings using image stimuli in Baron and Osherson 2011). Whether this reflects two different cognitive processes in the same brain region, i.e., both a simple co-activation of both components (captured by addition neural model) and an interactive process of composition (multiplication neural model), and whether there are other types of nonlinear operations (e.g., Wang and Zong 2017) of the neural mechanisms that better underlie the interactive compositional processes of word meaning, remain to be further tested.

### The role of the bilateral ATL in social composition

The findings that activation patterns of the bilateral ATL showed significant positive correlations with the social-person phrase similarity matrix (“hug mother”), i.e., that social phrases elicit more similar neural patterns than others, are in line with previous studies which showed effects of concept composition in ATL mostly using univariate analysis of adjective–noun (modifying) phrases (Bemis and Pylkkänen 2011; Pallier et al. 2011; Pylkkänen et al. 2014; Westerlund et al. 2015; Westerlund and Pylkkänen 2014). However, it is uncertain how such effects reflect “composition” in nature—the social phrases did not generate stronger activation than non-meaningful phrases; the phrase activation patterns were not predicted by the synthesis of the components; manipulation phrases (e.g., “fold napkin”) did not produce RSA similar effects. We speculate that the following issues may need to be considered in the future studies to understand this complex pattern across studies of phrase compositions in ATL. One is morphosyntactic structures of the composition (Flick et al. 2018), which requires more systematic manipulations of both semantic domains and morphosyntactic structures of the phrases. Second, it has been recently proposed that the relative semantic specifity of the concepts being combined (e.g., first word having stronger modification effects on the second) modulates the response pattern (Westerlund and Pylkkänen 2014; Zhang and Pylkkänen 2015; see review in Pylkkänen 2019). It is to be empirically tested whether social and manipulation domains differ systematically along this dimension. A third consideration is the manner of stimuli presentation. Some of these magnetoencephalography studies presented phrases word by word (rather than presenting on the same screen as in our study) and emphasized the ATL effects during the processing of a head noun that was modified by a previously presented modifier (Bemis and Pylkkänen 2011; Westerlund and Pylkkänen 2014). Nonetheless, it does not seem that the ATL effect in semantic composition is limited to the word-by-word stimulus presentation paradigm, given that Pylkkänen et al. (2014), using picture as stimuli, also found ATL composition effects in a production task (e.g., red tree versus red, blue).

Independent evidence has suggested ATL’s implication in social information processing in general. It has been consistently found to be sensitive to social concepts (Binney et al. 2016; Foley et al. 2020; Lin et al. 2018; Ross and Olson 2010; Wang et al. 2019; Zahn et al. 2007). Mellem et al. (2016) used an increased constituent paradigm (single word, 3-word phrase, and 6-word sentence) and found that the anterior portion of the left superior temporal gyrus responded to an increasing constituent structure with regard to social–emotional content. Zhang et al. (2021) found that social semantic networks, including the bilateral ATL showed stronger social accumulative effects using contrasts between sentence/narrative and word list reading. The current findings that ATL represents information about social phrases but does not respect the additive or multiplication synthetic models of the constituents, suggest that this region may be sensitive to the result of social semantics and not the combinational processes of social events.

A final methodological note is that we did not observe social-phrase-specific effects in a range of regions that have been previously implicated in social processing, including the bilateral posterior superior temporal sulcus, inferior frontal gyrus and dorsomedial prefrontal cortex. One possible explanation for this outcome is that the contrasting condition—manipulation of an object—is still performed by human agents and by one common definition is still social. Thus, the contrast may be stricter than those used in the literature showing social preference effects in these regions. This may also explain the overall negative findings of the manipulation-artifact condition.

## Conclusion

To conclude, we observed that the bilateral IOG, ITG, and ATL showed a preference for processing social-event semantic phrases, revealed by univariate analyses and multivariate RSA analyses. The right IOG and left ITG further showed a neural compositional effect, with stronger correlations between neural activation patterns observed for social-action-person phrases and the synthesis of the neural activation patterns of the constituent words, than that for the parallel correlations of manipulation phrases. No regions were observed showing evidence of phrase composition for both domains or stronger effects of manipulation phrases. These findings highlight the roles of the visual cortex and ATL in social-event processing, suggesting a domain-preference, rather than domain-general mechanisms of verbal event composition.

## Data Availability

The data that support the results of the present study are available from the corresponding author upon reasonable request.
